# Case Report: Rare spontaneous hepatic rupture hemorrhage complicated by autoimmune liver disease

**DOI:** 10.3389/fmed.2026.1767862

**Published:** 2026-03-25

**Authors:** Limin Ma, Changming Tao, Jichun Yang, Yingying Zhang, Yanan Qin

**Affiliations:** 1 Department of Infectious Diseases, Liaocheng People’s Hospital, Liaocheng, China; 2 Department of Laboratory Medicine, Dongchangfu District Hospital of Traditional Chinese Medicine, Liaocheng, China

**Keywords:** autoimmune hepatitis, hepatic rupture hemorrhage, overlap syndrome, perihepatic hematoma, primary biliary cholangitis

## Abstract

Autoimmune liver disease is predominantly characterized by autoimmune hepatitis and primary biliary cholangitis. In clinical practice, patients with autoimmune liver disease may progress to cirrhosis and subsequently develop multiple complications of decompensated cirrhosis, such as ascites, hepatic encephalopathy, and gastrointestinal hemorrhage. However, spontaneous hepatic rupture hemorrhage in patients with autoimmune liver disease is exceedingly rare. This article presents a case of presumed spontaneous hepatic rupture hemorrhage in a patient with autoimmune hepatitis-primary biliary cholangitis overlap syndrome and cirrhosis. Through multidisciplinary evaluation, conservative management was selected, achieving successful hemostasis. This report aims to provide a reference for the clinical recognition and management of such rare complications.

## Introduction

Autoimmune liver disease (AILD) is an inflammatory injury of the hepatobiliary system mediated by aberrant autoimmune responses, primarily encompassing autoimmune hepatitis (AIH), characterized by predominant hepatocellular damage, as well as primary biliary cholangitis (PBC) and primary sclerosing cholangitis (PSC), both featuring biliary injury and cholestasis as hallmark manifestations (1, 2). Some AILD cases exhibit overlapping clinical or pathological features of both AIH and PBC (or PSC), constituting what is termed overlap syndrome (OS), with AIH-PBC OS being the most prevalent subtype. AILD patients carry a risk of progression to liver cirrhosis, with some cases advancing rapidly to the decompensated stage at initial presentation (3). Liver transplantation remains the sole effective therapeutic intervention for prolonging survival and improving quality of life (4). Patients with decompensated cirrhosis frequently develop multiple complications, including esophageal and gastric variceal bleeding, ascites, spontaneous bacterial peritonitis, and hepatic encephalopathy. However, spontaneous hepatic rupture hemorrhage in patients with AILD is clinically rare. Spontaneous hepatic rupture refers to parenchymal tearing without identifiable external trauma, constituting a surgical emergency that may rapidly become life-threatening. Clinical case reports indicate hepatic rupture most commonly occurs in hepatocellular carcinoma (HCC) or hepatic hemangioma rupture (5), whereas perihepatic hematoma or hepatic rupture associated with AILD is exceedingly scarce in the literature. We present the diagnostic and therapeutic management of a case involving perihepatic hematoma (presumed spontaneous hepatic rupture) in an AIH-PBC OS patient with cirrhosis. The successful conservative management experience offers valuable insights for analogous cases.

## Case presentation

A 47-year-old male patient was admitted to the Hepatology Department of Liaocheng People’s Hospital on March 16, 2025, presenting with “AILD diagnosed two years prior and abdominal pain persisting for 20 hours.” Two years ago, the patient was hospitalized at the same institution for “fatigue and dark urine.” Comprehensive serological evaluation revealed a negative anti- mitochondrial M2 antibody, a strongly positive gp210 (+++), and equivocal RO-52 (±). Liver histopathology (light microscopy) demonstrated pseudolobule formation, diffuse hydropic degeneration and ballooning degeneration of hepatocytes, cholestasis in hepatocytes and canaliculi, scattered spotty and confluent necrosis, and intracytoplasmic pigment deposition. Hepatic sinusoids exhibited extensive mixed inflammatory infiltration predominantly composed of mononuclear cells, including segmented leukocytes, plasma cells, and Kupffer cells. Expanded portal tracts featured fibrous hyperplasia with septa formation, prominent bile duct proliferation, vacuolar degeneration and loss of interlobular bile duct epithelium, and moderate interface hepatitis. These findings supported a diagnosis of autoimmune hepatitis-AIH-PBC OS, classified as G3S4 with mild intrahepatic cholestasis. Immunohistochemistry showed CK19 (bile ducts +), CK7 (bile ducts +), Mum-1 (focal +), CD68 (focal +), and CD34 (vasculature +). Special staining confirmed MASSON trichrome (+) and reticulin fiber (+). The definitive diagnosis of AIH-PBC OS was established through integrated clinicopathological assessment. Therapeutic management included mycophenolate mofetil capsules (0.5 g bid), calcium carbonate D3 chewable tablets (III), calcitriol soft capsules, adenosylmethionine enteric-coated tablets, methylprednisolone tablets (4 mg qd), ursodeoxycholic acid capsules (500 mg bid), esomeprazole magnesium enteric-coated tablets, diammonium glycyrrhizinate, and spironolactone. The patient required subsequent hospitalizations for cirrhosis complicated by ascites.

**Figure 1:**
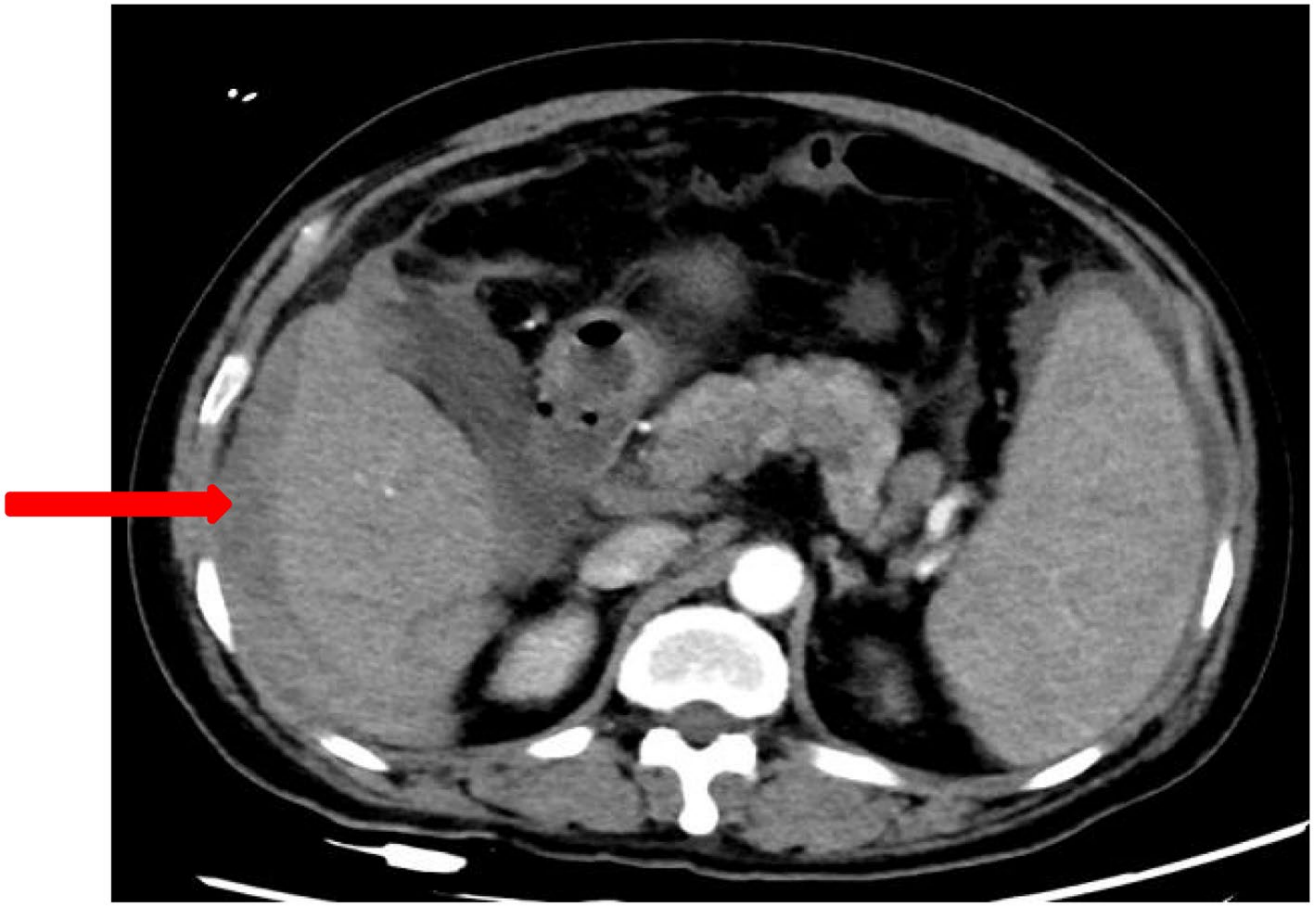
Contrast-enhanced CT during acute presentation. Key features: ① perihepatic hematoma (arrowheads) with homogeneous attenuation (50–79 HU), ② nodular liver surface without capsular discontinuity, ③ absence of active extravasation or parenchymal laceration, ④ ascitic fluid (asterisk), ⑤ splenomegaly, ⑥ enlarged perigastric lymph node, ⑦ dilated esophagogastric collateral vein.

**Figure 2:**
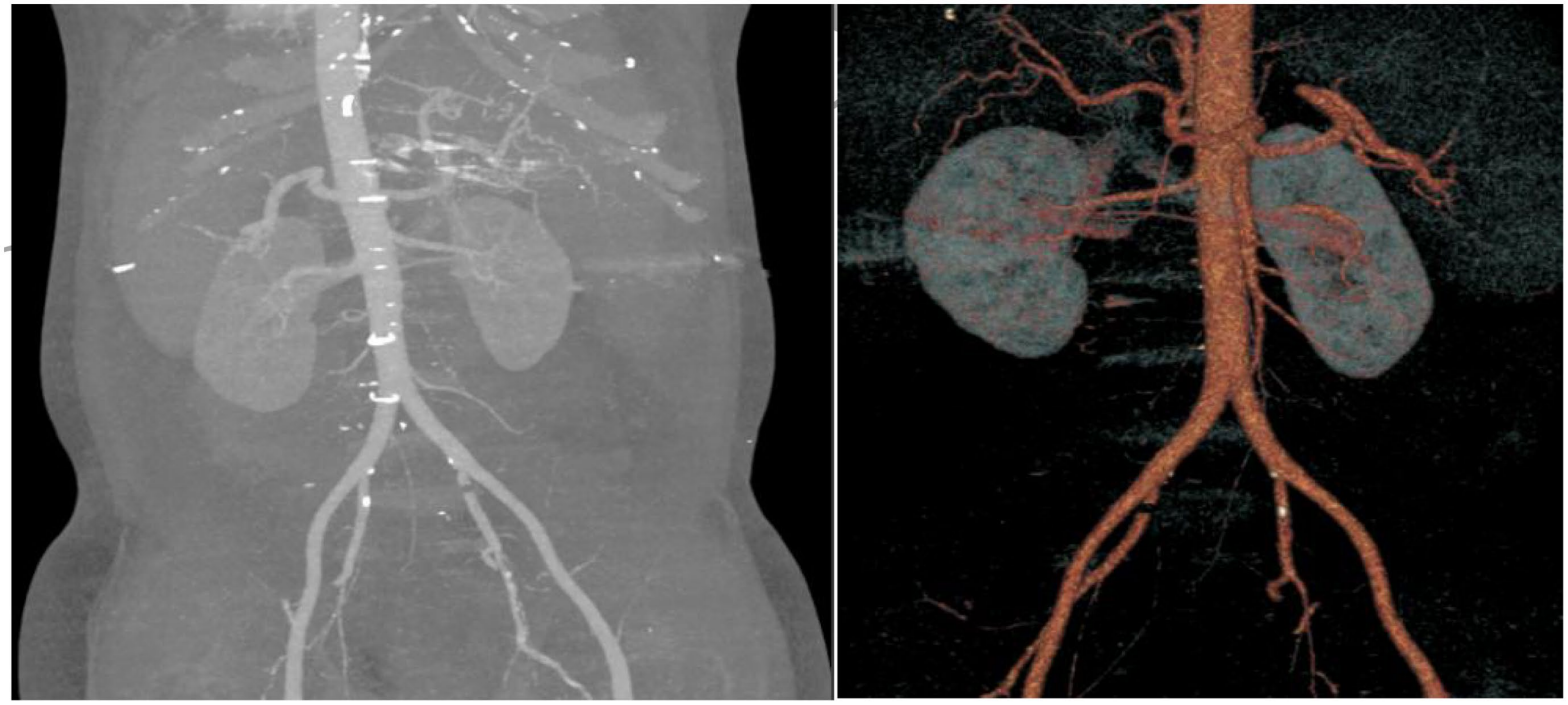
Abdominal CTA in the arterial phase reveals no evidence of contrast extravasation or vascular abnormality in the abdominal aorta, bilateral renal arteries, celiac trunk, or superior and inferior mesenteric arteries and their branches.

Upon current admission, the patient developed persistent, needle-like pain in the right upper quadrant 20 h prior without identifiable triggers. The pain was position-independent, exacerbated postprandially, and accompanied by nausea. No vomiting, diarrhea, fever, chest tightness, shortness of breath, dizziness, headache, or melena was reported. Blood tests showed a red cell count (RBC) of 2.18 × 10^12^/L, hemoglobin (HGB) of 69 g/L, hematocrit (HCT) of 0.223 L/L, platelet (PLT) count of 98 × 10^9^/L, and C-reactive protein (CRP) at 105 mg/L. Contrast-enhanced computed tomography of the upper abdomen demonstrates cirrhotic liver morphology, characterized by a nodular contour and heterogeneous parenchymal enhancement. A perihepatic hematoma (arrowheads) is observed in the right subphrenic space, with attenuation values ranging from 50 to 79 HU, and no evidence of active contrast extravasation (see Figure 1). The hematoma appears homogeneous and is not associated with an underlying mass or capsular disruption. No focal parenchymal laceration or traumatic injury pattern was identified. Additional findings include splenomegaly, ascites, indistinct peritoneal interfaces, multiple enlarged lymph nodes along the lesser gastric curvature and superior to the pancreatic head, cholecystitis, and tortuous dilated veins in the esophagogastric and splenic hilar regions. Abdominal computed tomography angiography (CTA) showed that arterial phase showed normal filling of the abdominal aorta, bilateral renal arteries, celiac trunk, superior/inferior mesenteric arteries and their branches without abnormal densities (Figure 2). Diagnostic paracentesis in the emergency department yielded non-coagulable blood. Hemostatic agents, including etamsylate and thrombin were administered emergently. The patient was admitted to the hepatology unit with a presumptive diagnosis of “perihepatic hematoma, presumed hepatic rupture.” Past medical/social history: Denied chronic conditions including hypertension, diabetes, and cardiac or cerebrovascular diseases. No history of smoking or alcohol abuse. Unremarkable personal and family histories.

Physical examination findings on admission included: pulse (P) 82 beats/min and blood pressure (BP) 102/61 mmHg. The patient was alert but lethargic, with an anemic appearance and hepatic facies. Icteric sclera and mucous membranes were noted, without petechiae. Pupils were isocoric, and the light reflex was prompt. Coarse breath sounds were heard bilaterally without rales. Cardiac rhythm was regular with strong heart sounds: no murmurs were detected. The abdomen was mildly distended and doughy on palpation, without palpable masses. Tenderness was elicited in the right upper quadrant without rebound tenderness. The liver was not palpable below the xiphoid or costal margin. The spleen was palpable 5 cm below the left costal arch. Murphy’s sign was negative, and McBurney’s point was non-tender. No costovertebral angle tenderness. Shifting dullness was absent. Bowel sounds were normal. Mild bilateral lower extremity edema was present.

### Complementary examinations

Liver function tests showed levels of alanine aminotransferase (ALT) of 30 U/L, aspartate aminotransferase (AST) of 71 U/L, alkaline phosphatase (ALP) of 333 U/L, Y-glutamyltranspeptidases (y-GT) of 78 U/L, and total bilirubin (TBIL) of 164.7 μmol/L. Kidney function assessment demonstrated a serum creatinine level of 42 μmol/L and blood urea nitrogen (BUN) of 4.04 mmol/L. Coagulation studies revealed prothrombin time (PT) of 15.1 s, prothrombin percentage activity (PTA) of 75%, international normalized ratio (INR) of 1.15, and D- dimer levels of 8.65 mg/L. Ascites analysis showed that Reddish, turbid, non-coagulating (hemorrhagic); Rivalta test (−); nucleated cell count of 24,200 × 10^6^/L.

### Treatment and regression

Strict bed rest, supplemental oxygen via nasal cannula, volume expansion, etamsylate (2 g q12h IV), hemocoagulase agkistrodon (2 U q8h IV), magnesium isoglycyrrhizinate (150 mg qd IV), adenosylmethionine (1 g qd IV), and piperacillin/tazobactam (4.5 g q8h IV). The multidisciplinary consultation consensus indicated that the perihepatic hematoma originated from a presumed spontaneous hepatic rupture. Given the patient’s hemodynamic stability and limited hemorrhage on imaging, conservative management with close observation was recommended. An upper abdominal MRI showed resolution of perihepatic hematoma with minimal residual fluid 12 days later. Blood tests showed a red cell count (RBC) of 2.82 × 10^12^/L, hemoglobin (HGB) of 92 g/L, and C-reactive protein (CRP) at 36.87 mg/L. Liver function tests showed levels of alanine aminotransferase (ALT) of 45 U/L, aspartate aminotransferase (AST) of 64 U/L, alkaline phosphatase (ALP) of 383 U/L, Y-glutamyltranspeptidases (y-GT) of 98 U/L, and total bilirubin (TBIL) of 96.7 μmol/L. Coagulation studies revealed a prothrombin time (PT) of 14.2 s, prothrombin percentage activity (PTA) of 76%, an international normalized ratio (INR) of 1.09, and D-dimer levels of 2.5 mg/L. Significant clinical improvement was noted (Table 1). Patient discharged.

**Table 1:** Timeline of key laboratory values and imaging findings.

Time point	Key laboratory values	Key imaging findings
Hospital day 1	Hemoglobin 69 g/L, platelet 98 × 10^9^/L, prothrombin time 15.1 s, international normalized ratio 1.15, prothrombin percentage activity 75%, D-dimer 8.65 mg/L, total bilirubin 164.7 μmol/L	Contrast-enhanced CT of the upper abdomen disclosed cirrhosis with perihepatic hematoma (CT value 50–79 HU); splenomegaly, ascites, and blurred peritoneal interfaces; indistinct hepatic inferior margin; multiple enlarged lymph nodes along the lesser gastric curvature and superior to the pancreatic head; cholecystitis; tortuous dilated veins in the esophagogastric and splenic hilar regions. Abdominal CTA showed that arterial phase showed normal filling of the abdominal aorta, bilateral renal arteries, celiac trunk, superior/inferior mesenteric arteries and their branches without abnormal densities
Hospital day 2	Hemoglobin 72 g/L, platelet 108 × 10^9^/L	
Hospital day 5	Hemoglobin 73 g/L, platelet 92 × 10^9^/L	
Hospital day 13/before discharge	Hemoglobin 92 g/L, platelet 141 × 10^9^/L, prothrombin time 14.2 s, international normalized ratio 1.09, prothrombin percentage activity 76%, D-dimer 2.5 mg/L, total bilirubin 96.7 μmol/L	Contrast-enhanced MRI of the abdomen reveals liver cirrhosis, splenomegaly, ascites, and the presence of esophagogastric and splenic varices. There is an abnormal signal along the inferior hepatic margin and diffuse heterogeneous signal intensity within the liver parenchyma, suggestive of cirrhotic nodules
6-month post-discharge	Hemoglobin 108 g/L, platelet 154 × 10^9^/L, prothrombin time 17.2 s, international normalized ratio 1.19, prothrombin percentage activity 68%, D-dimer 3.62 mg/L, total bilirubin 56.6 μmol/L	Abdominal CT shows liver cirrhosis, dilation of the portal and splenic veins, splenomegaly, and ascites

### Follow-up

The patient completed a 6-month structured follow-up. Patient-assessed outcomes indicated complete resolution of abdominal pain. Follow-up diagnostic imaging included contrast-enhanced abdominal CT performed at 2 and 6 months post-discharge, demonstrating with complete resolution of the previous subcapsular hematoma and no signs of recurrence. Laboratory evaluation showed gradual improvement in hepatic synthetic function, with serum albumin increasing from 28 g/L at discharge to 35 g/L at 6 months, and normalization of coagulation parameters. The patient demonstrated full adherence to prescribed medical therapy without reported tolerability issues. No adverse events, unanticipated complications, or readmissions occurred during the follow-up period.

## Discussion

This report documents a case of a patient diagnosed with AIH-PBC OS who progressed to decompensated cirrhosis and developed a perihepatic hematoma. After multidisciplinary evaluation, the hematoma was determined to be due to presumed spontaneous hepatic rupture. This case highlights that, although extremely rare, such critical complications may occur in patients with advanced AILD, particularly those with concomitant cirrhosis and coagulopathy. The successful hemostasis achieved thru conservative treatment in this patient provides valuable reference for the management of similar clinical scenarios and has significant implications for non-interventional therapeutic plans.

Spontaneous hepatic rupture hemorrhage in patients with AILD is clinically rare. The underlying pathogenesis remains incompletely elucidated but is predominantly linked to the consequences of cirrhosis and coagulopathy. Chronic, recurrent inflammation culminates in fibrosis and cirrhosis. Fibrotic tissue exhibits inherent stiffness and heterogeneity, while structural disparities between regenerative nodules and fibrotic septa increase hepatic fragility. Concurrent portal hypertension induces vascular dilation and congestion within the liver and subcapsular regions, elevating vascular wall tension and fragility. Against this backdrop of structural vulnerability, portal hypertension further amplifies the risk of vascular rupture. Coagulopathy in cirrhosis (reduced clotting factor synthesis, thrombocytopenia) also impairs effective hemostasis post-rupture (6, 7). Alternative explanations for spontaneous hemorrhage warrant consideration: subcapsular or perihepatic hematoma without definitive rupture: This entity typically presents with contained hemorrhage and is often amenable to conservative management in stable patients, similar to the approach taken here. However, the diagnostic paracentesis of this case revealed non-coagulable bloody fluid, indicating true capsule rupture rather than localized hematoma. Portal hypertension-related bleeding (small vessel rupture due to vascular congestion): While portal hypertension is a known risk factor for spontaneous hemorrhage, bleeding solely from congested small vessels without parenchymal disruption might be expected to cause a less abrupt presentation. The focal nature of the hemorrhage adjacent to the liver periphery, rather than a diffuse pattern, and the absence of dominant portal collaterals at the bleeding site. Coagulopathy-induced microvascular leakage: Diffuse capillary hemorrhage secondary to thrombocytopenia/factor deficiency. However, diffuse microvascular leakage secondary to coagulopathy alone would typically manifest more diffusely rather than as a focal hemorrhage adjacent to the liver surface. Non-parenchymal sources: Splenic or mesenteric variceal rupture masquerading as hepatic hemorrhage. These alternatives cannot be definitively excluded given the limitations of cross-sectional imaging in differentiating microvascular events from true parenchymal rupture. The presenting anemia (Hb 69 g/L) and coagulopathy (prolonged PT, elevated D-dimer) in this case underscore the significant contribution of coagulopathy. While autoimmune inflammation may theoretically contribute to liver fragility (8), its specific role in rupture pathogenesis remains speculative and less well-defined compared to the paramount factors of cirrhosis and coagulopathy. Further studies are needed to clarify whether autoimmune mechanisms independently increase rupture risk beyond cirrhosis-related factors.

Traumatic injury constitutes the predominant etiology of hepatic rupture. Patients with underlying hepatomegaly—such as in amyloidosis, malaria, hepatic congestion, or hepatic neoplasms—are particularly susceptible to traumatic rupture upon external impact. In contrast, spontaneous hepatic rupture occurring without identifiable trauma is relatively rare yet clinically critical, with high mortality rates due to rapid progression to hemorrhagic shock if not promptly recognized and managed (9). Among spontaneous hepatic rupture cases, HCC overwhelmingly predominates as the leading cause (10). A comprehensive review by Chen et al. (9) analyzing 70 cases of spontaneous hepatic rupture in China identified HCC as the primary etiology (85.7%), followed by hepatic adenoma (5.7%), cirrhosis (4.3%), hemangioma (2.9%), and metastatic liver tumors (2.9%). Spontaneous hepatic rupture in patients with AILD, particularly non-neoplastic forms, is exceedingly uncommon, making the present case involving AIH-PBC OS a notable addition to the sparse literature.

Spontaneous hepatic rupture typically presents with acute abdominal pain, nausea, vomiting, hemodynamic instability, and rapid hemoglobin decline, other laboratory tests also include elevated liver enzymes, coagulopathy, and thrombocytopenia, etc. (11–13). The diagnosis must be confirmed thru imaging to identify liver parenchyma destruction or active bleeding. Enhanced computed tomography (CT) has become the preferred diagnostic method due to its high sensitivity and specificity (14). CT findings include active contrast extravasation, hematoma (usually 35–45 HU), irregular liver contour (indicating capsule rupture), and intraparenchymal or subcapsular hematomas (14). Angiographic findings such as arterial jet, microleakage, or capillary leakage are diagnostic when present, but this method is primarily used for therapeutic intervention rather than diagnosis (14). The presence of active extravasation on CT can predict the failure of conservative treatment, strongly indicating the need for transcatheter arterial embolization (TAE), which can be performed during the same angiography procedure. Therefore, CT serves as the gatekeeper for diagnosis, while angiography plays a supportive role in achieving hemostasis and avoiding emergency surgery (15). Spontaneous hepatic rupture carrying a high risk of hemorrhagic shock and mortality, underscoring the critical importance of early recognition. Most cases necessitate urgent intervention to control bleeding. For hemodynamically unstable patients, transcatheter arterial embolization (TAE) is often prioritized as a minimally invasive technique to achieve rapid hemorrhage control, particularly in HCC-associated cases. Subsequent elective surgery may be considered upon stabilization (10). Our patient manifested classic features, with contrast-enhanced CT confirming contained subcapsular hematoma without active extravasation. Considering that the patient’s bleeding is limited to the perihepatic area and hemodynamics remain stable, after a multidisciplinary consultation and detailed communication with the patient’s family to seek their consent, it was decided not to perform digital subtraction angiography at this time and to choose a conservative treatment plan. During the treatment period, the patient’s symptom relief, vital signs, and hemoglobin levels were strictly monitored, and imaging examinations confirmed the improvement of the condition, ultimately achieving successful hemostasis. This outcome highlights the importance of individualized therapeutic decision-making, demonstrating that conservative management can be a viable option in highly selected, stable patients with contained hemorrhage. This contrasts with the majority of reported cases, where active bleeding or instability necessitated intervention (8, 10, 16).

### Limitations

It is necessary to clearly point out the following limitations: Diagnostic certainty: As stated throughout the text, due to the lack of definitive evidence from hepatic arteriography or surgical exploration, the diagnosis of liver rupture remains presumptive. Although the clinical presentation and CT findings are highly indicative, the lack of direct imaging or angiographic evidence constitutes an inherent limitation of the retrospective case report format and the clinical decision-making for this unstable patient. Limitations of case reports: As a single case report, this study cannot establish causality or generalize the research findings. The rarity of spontaneous liver rupture in AILD makes it difficult to draw broader conclusions. The observations are only applicable to the unique clinical context of this patient. Pathophysiological specificity: Although cirrhosis and coagulopathy have been emphasized as primary risk factors, the specific contribution of the autoimmune inflammatory process (AIH-PBC OS) to the rupture risk in this patient, aside from causing cirrhosis, remains speculative and cannot be definitively determined based solely on this case.

## Conclusion

This report provides a detailed account of the diagnosis and management of a perihepatic hematoma (presumed spontaneous hepatic rupture) in a patient with cirrhosis secondary to AIH-PBC OS. Its core contributions include: (1) Documenting a case of presumed spontaneous hepatic rupture in a patient with concomitant AILD against the background of cirrhosis with coagulopathy, reinforcing that this complication may arise in diverse etiologies of advanced liver disease; (2) Demonstrating the feasibility and effectiveness of conservative treatment for specific patients (hemodynamically stable and with limited hemorrhage) after urgent identification thru multidisciplinary evaluation (hepatology, hepatobiliary surgery, interventional radiology); (3) Emphasizing that in chronic liver disease patients (especially those with decompensated cirrhosis) presenting with acute upper abdominal pain, hepatic rupture must be a key differential diagnosis and immediate enhanced CT examination is necessary to confirm the diagnosis.

## Data Availability

The original contributions presented in the study are included in the article/supplementary material, further inquiries can be directed to the corresponding authors.
